# Effectiveness and Safety of Risankizumab for Psoriasis in a Liver Transplant Patient With Recurrent Metastatic Hepatocellular Carcinoma

**DOI:** 10.7759/cureus.89494

**Published:** 2025-08-06

**Authors:** Sandy Sylvain, Christine Silvain, Matthieu Bainaud, Thomas Kerforne, Marie Masson Regnault

**Affiliations:** 1 Dermatology, Poitiers University Hospital, Poitiers, FRA; 2 Gastroenterology, Poitiers University Hospital, Poitiers, FRA; 3 Oncology, Poitiers University Hospital, Poitiers, FRA; 4 Organ and Tissue Procurement Coordination, Poitiers University Hospital, Poitiers, FRA; 5 Dermatology, Hôpital Privé Francheville, Perigueux, FRA

**Keywords:** biological therapy, cancer, psoriasis, risankizumab, severe renal impairment, transplant organ

## Abstract

Risankizumab (RZB) is a humanized monoclonal antibody that selectively targets interleukin-23 (IL-23). It has proven particularly effective in treating psoriasis, a common chronic inflammatory skin disease. However, its use remains poorly documented in certain populations, including patients with a history of solid organ transplantation or recent/active malignancy. We present a case of successful and well-tolerated treatment with RZB over an 18-month follow-up period in a patient with multiple comorbidities, including liver transplantation, recurrent hepatocellular carcinoma (HCC) with adrenal metastases, and severe chronic kidney disease.

## Introduction

Risankizumab (RZB) is a humanized monoclonal antibody that binds with high affinity to the p19 subunit of interleukin-23 (IL-23). It has demonstrated high efficacy in the treatment of psoriasis, a common chronic inflammatory skin disease. However, managing psoriasis in patients who have undergone solid organ transplantation presents a significant therapeutic challenge, as it requires balancing the immunosuppression needed for graft survival with the control of an immune-mediated inflammatory condition. In this population, the use of biologic therapies is often limited by the risk of infection, potential drug interactions, and uncertainties surrounding oncologic safety. To date, the use of biologics has been scarcely studied in specific subpopulations such as transplant recipients and individuals with active or recently treated cancer. Their use in these groups raises concerns due to the potential for enhanced immunosuppression and increased risk of tumor recurrence. Here, we report a rare case of RZB treatment in a liver transplant recipient who recently experienced a recurrence of hepatocellular carcinoma (HCC).

## Case presentation

**Figure 1:**
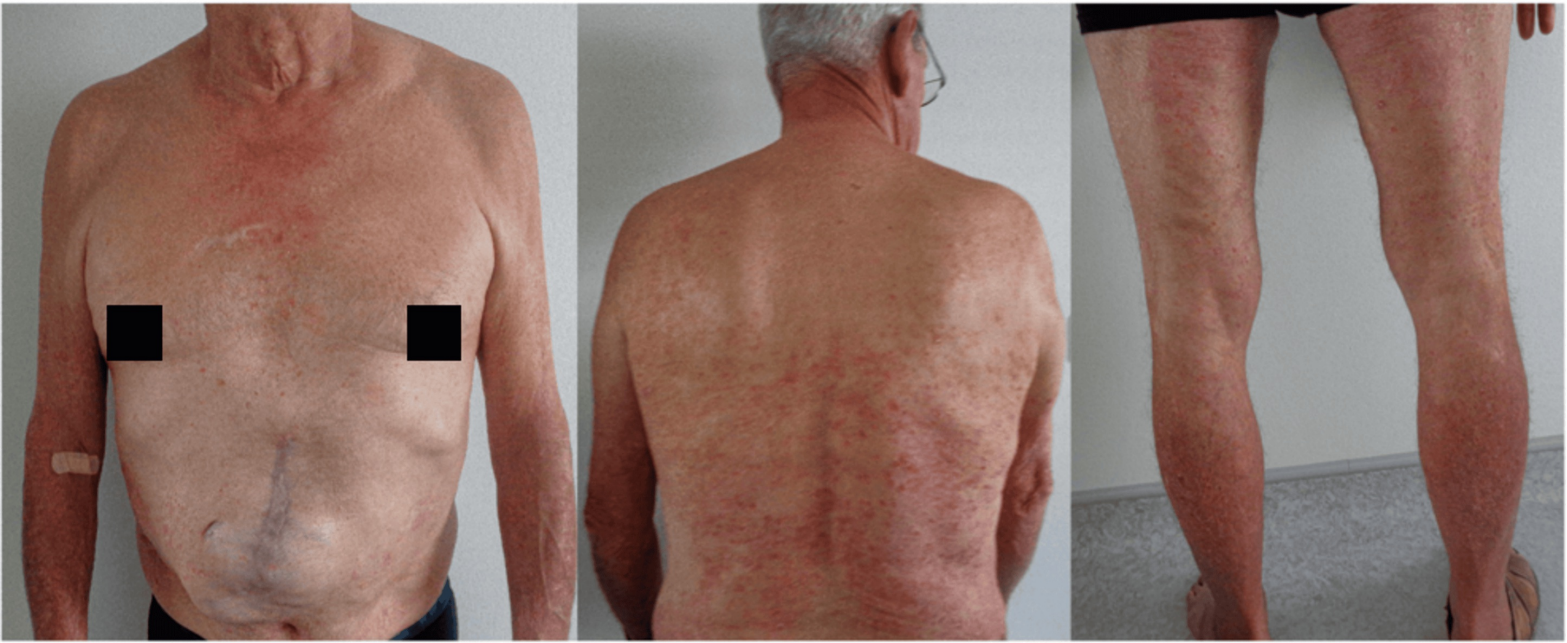
Clinical presentation of psoriasis before initiation of the treatment

**Figure 2:**
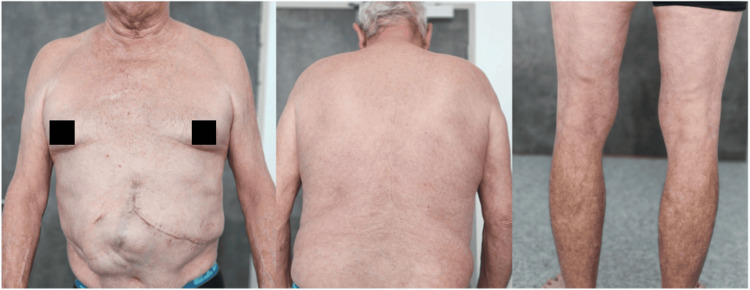
Clinical presentation after three months of treatment

A 65-year-old man was referred to the dermatology department for severe psoriasis, which had been present since 1997 and had worsened over the previous two months. His medical history included a liver transplant in 2022 for HCC in the context of alcoholic and metabolic cirrhosis, treated with oral tacrolimus. He also had type II diabetes and severe chronic kidney disease (creatinine clearance: 20 mL/min). A recent recurrence of HCC with adrenal metastasis had been treated by complete surgical excision less than one month prior. No adjuvant therapy was given, and no recurrence had been observed since the intervention. Over the past two months, the patient showed worsening psoriasis, with a body surface area (BSA) involvement of 30%, a Psoriasis Area and Severity Index (PASI) score of 12.4, and a Physician’s Global Assessment (PGA) of 4 (Figure 1). Previous treatment with acitretin had yielded poor efficacy and tolerance. Methotrexate, cyclosporin, and narrow-band ultraviolet light were contraindicated due to his liver transplant, renal failure, and recent metastatic disease. Given these limitations, initiation of biologic therapy was proposed. RZB was selected in agreement with the transplant team, hepatologist, and oncologist. After 16 weeks of treatment, the patient achieved complete clearance of skin lesions (BSA: 0%, PASI: 0, PGA: 0) (Figure 2). No adverse effects were reported, and liver and kidney function tests remained stable. At 18 months following treatment initiation, there was no evidence of graft rejection or cancer recurrence. The treatment remained highly effective and well tolerated (BSA: 0%, PASI: 0, PGA: 0).

## Discussion

Management of moderate-to-severe psoriasis in organ transplant patients is particularly challenging. According to the recommendations of the American Academy of Dermatology, first-line treatment includes acitretin, with or without narrow-band ultraviolet light [1]. Second-line therapy involves increasing the dose of immunosuppressive agents. Other systemic or biologic treatments are generally reserved for more severe or refractory cases [1]. The use of biologic therapies in transplant recipients remains largely undocumented. To our knowledge, only 19 cases have been reported in the literature, including three treated with RZB. Other agents used were etanercept (n = 7), adalimumab (n = 2), ustekinumab (n = 2), brodalumab (n = 3), and ixekizumab (n = 2) (Table 1). In all cases, the treatments were effective and well tolerated, with follow-up durations ranging from 6 months to 5 years [2,3]. The IL-17/IL-23 axis (TH17 pathway) has also been implicated in the pathophysiology of transplant rejection, suggesting that IL-17 and IL-23 may be promising therapeutic targets for preventing immunological complications in this setting [4]. Our case is further distinguished by the patient's recent history of cancer recurrence. While a recent meta-analysis of 17 clinical trials found no increased risk of malignancy with RZB use [5], the question of cancer recurrence during treatment remains unanswered. Limited data are available on this subject. One retrospective multicenter Italian study evaluated the safety of biologics in psoriatic patients with a recent cancer diagnosis. Twenty-six patients initiated biologic therapy five years after their cancer diagnosis (secukinumab, n = 12; ixekizumab, n = 2; guselkumab, n = 1; etanercept, n = 3; ustekinumab, n = 4; brodalumab, n = 1), and no recurrences were reported after a mean follow-up of 30.69 ± 22.24 months [6]. Additionally, preclinical studies have shown that elevated IL-23 levels are associated with poorer prognosis in various cancers. Based on these findings, some authors have proposed targeting IL-23p19 and downstream pathways as a potential strategy to reduce tumor burden and inhibit metastasis [7,8].

**Table 1:** Characteristics of transplanted patients treated with biologic therapies for psoriasis *Asymptomatic urinary tract infections and recurrent cholangitis (history of recurrent cholangitis before etanercept). Source: [2,3,8-20].

Author	Age, sex	Transplanted organ	Immunosuppressive regimen	Immunobiological	Time of use (follow-up)	Clinical response	Complications
Present study	65, M	Liver	Advagraf	Risankizumab	September 2023 to present	Yes (PASI 100: 4 months)	None
Luz et al. (2024) [3]	49, M	Liver	Tacrolimus, mycophenolate mofetil	Risankizumab	14.5 months	Yes (PASI 100: 4 months)	None
Luz et al. (2024) [3]	55, M	Kidney	Tacrolimus, mycophenolate mofetil, prednisone	Risankizumab	10 months	Yes (PASI 100: 4 months)	None
Meneghello et al. (2023) [2]	39, M	Kidney	Tacrolimus, prednisone	Risankizumab	2021 to May 2023	Yes (PASI 90: 4 months)	None
Meneghello et al. (2023) [2]	45, M	Liver	Tacrolimus, mycophenolate mofetil	Etanercept	No data	Yes (PASI 90: 3 months)	None
Madankumar et al. (2015) [8]	52, M	Liver	Tacrolimus, prednisone, everolimus	Etanercept	12 months	Yes (PASI 90: 1 month)	Multiple infections*
Brokalaki et al. (2012) [9]	42, M	Pancreas, kidney	Prednisone, tacrolimus, mycophenolate mofetil	Etanercept	24 months	Yes (PASI 90: 1 month)	None
Hoover (2007) [10]	63, M	Liver	Tacrolimus, sirolimus	Etanercept	12 months	Yes (PASI 60: 1.5 months)	None
Collazo et al. (2008) [11]	49, M	Liver	Tacrolimus, mycophenolate mofetil, corticosteroids	Etanercept	5 months	Yes (PASI 100: 3 months)	None
García-Zamora et al. (2020) [12]	67, M	Kidney	Tacrolimus, mycophenolate mofetil, corticosteroids	Etanercept	10 months	Yes (PASI 90: 3 months)	None
De Simone et al. (2016) [13]	56, M	Liver	Prednisone, tacrolimus, mycophenolate mofetil	Etanercept	6 months to re-starting after recurrence	Yes (PASI 90: 3 months)	None
Blasco et al. (2024) [14]	29, M	Kidney	Prednisolone, tacrolimus	Adalimumab	42 months	Yes	None
Guglielmo et al. (2024) [15]	60, M	Liver	Tacrolimus, mycophenolate mofetil	Adalimumab	12 months	Yes	Facial warts, paranasal sinus mycetoma
Meneghello et al. (2023) [2]	53, M	Kidney	Sirolimus, acitretin	Ustekinumab	48 months	Yes (PASI 90: 2 months)	None
Richetta et al. (2021) [16]	50, M	Liver	Tacrolimus, mycophenolate mofetil	Ustekinumab	6 months	Yes (PASI 100: 2 months)	None
Singh et al. (2021) [17]	50, M	Liver	Tacrolimus, mycophenolate mofetil	Brodalumab	6 months	Yes (PASI 100: 2.5 months)	None
Matcaşu et al. (2023) [18]	71, M	Liver	Tacrolimus, mycophenolate mofetil	Brodalumab	12 months	Yes (PASI 100: 3 months)	None
Matcaşu et al. (2023) [18]	53, M	Kidney	Tacrolimus, mycophenolate mofetil, prednisolone	Brodalumab	5 months	Yes (PASI 100: 3 months)	None
Lora et al. (2019) [19]	54, M	Liver	Tacrolimus, mycophenolate mofetil	Ixekizumab	24 months	Yes (PASI 100: 3 months)	None
Di Altobrando et al. (2020) [20]	35, M	Kidney	Anti-thymocyte globulins, prednisone, tacrolimus, mycophenolic acid	Ixekizumab (patient under ixekizumab at the time of transplant)	10 months	Yes	None

## Conclusions

In conclusion, we report a case of successful and well-tolerated use of RZB for severe psoriasis in a patient with multiple comorbidities, including a history of liver transplantation, recent recurrence of HCC with adrenal metastasis, and severe chronic renal impairment. Sharing clinical experiences in managing such complex cases is crucial for developing safe, evidence-based guidelines for the treatment of moderate-to-severe psoriasis in transplant recipients and patients with a recent history of cancer.
